# Synergistic augmentation of osimertinib‐induced autophagic death by proguanil or rapamycin in bladder cancer

**DOI:** 10.1002/mco2.236

**Published:** 2023-04-29

**Authors:** Di Xiao, Simeng Xu, Xiaochen Zhou, Duo Li, Mei Peng, Xuetong Chu, Zhirong Zhang, Yan Peng, Alex F. Chen, Xiaoping Yang

**Affiliations:** ^1^ Key Laboratory of Study and Discovery of Small Targeted Molecules of Hunan Province Key Laboratory of Chemical Biology & Traditional Chinese Medicine Research of Ministry of Education Department of Pharmacy School of Medicine Hunan Normal University Changsha Hunan China; ^2^ Key Laboratory of Protein Chemistry and Developmental Biology of Fish of Ministry of Education Hunan Normal University Changsha Hunan China; ^3^ Institute for Developmental and Regenerative Cardiovascular Medicine Xinhua Hospital School of Medicine Shanghai Jiao Tong University Shanghai China

Dear Editor,

According to the latest global regional cancer data statistics, bladder cancer (BC) is the fourth most common cancer in men, and its mortality is also among the top 10 of all cancers.^1^ At present, the major treatment for BC is cystectomy. However, the cystectomy is difficult to perform in patients with unresectable or metastatic BC. PD‐1/PD‐L1 inhibitors and FGFR3 inhibitors have been approved for the clinical treatment of unresectable or metastatic BC, but their side effects and drug resistance of them have largely limited their effect.^2^ Thus, exploring new targeted therapies for BC has been an urgent clinic demand. Previous research has determined that EGFR is highly expressed in BC,^2^ indicating that EGFR may be an effective target for the treatment of BC. However, the use of first‐generation EGFR TKIs including erlotinib and monoclonal antibodies including cetuximab to treat BC patients in clinical trials have not shown the desired therapeutic effect.^2^, ^3^ Therefore, it is necessary to explore more effective targeted drugs to treat BC patients.

In this study, we found that osimertinib, the latest third‐generation EGFR inhibitor, significantly inhibited BC cell proliferation, colony formation and the expression of EGFR and its downstream signaling pathways (Figure 1A,B and Figure S1A–D), indicating that osimertinib may be a potential targeted drug for the treatment of BC.

**FIGURE 1:**
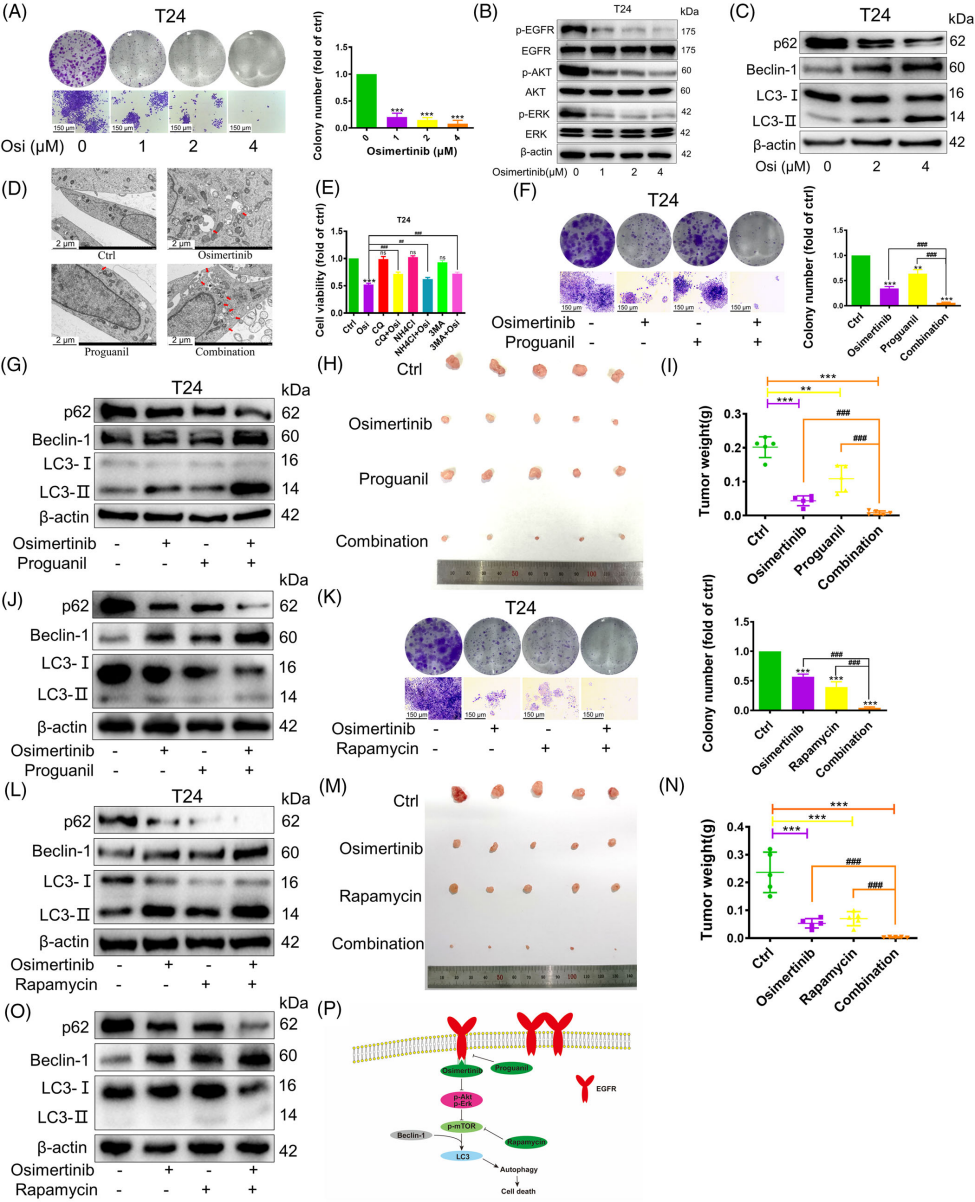
Autophagy induced by proguanil or rapamycin enhances antitumor effect of osimertinib in bladder cancer. (A) The effect of osimertinib (Osi) inhibiting T24 proliferation was evaluated using colony formation assay. Above: The full view of wells was taken through stereomicroscope. Below: A representative image of the well was taken through an inverted microscope. (B) T24 were treated with osimertinib for 24 h, and the expression of total or phosphorylated EGFR, AKT, and ERK were measured by Western blot. (C) T24 were treated with osimertinib for 24 h, and the expression of p62, Beclin‐1, and LC3 was measured by Western blot. (D) T24 were treated with osimertinib (2 μM) or proguanil (10 μM) for 24 h. Subsequently, cells are then fixed with electron microscope fixative and observed under a transmission electron microscope (red arrows indicate autophagosomes). (E) T24 were co‐treated with 5 μM CQ, 2 mM NH4Cl or 5 mM 3MA, and 4 μM osimertinib for 72 h, and the cell viability was measured by MTT. (F) T24 were treated with osimertinib (1 μM) and proguanil (5 μM) alone or in combination, and the cell viability was measured by colony formation assay. Above: The full view of wells was taken through stereomicroscope. Below: A representative image of the well was taken through an inverted microscope. (G) T24 were treated with osimertinib (2 μM) and proguanil (10 μM) for 24 h, and the expression of p62, Beclin‐1, and LC3 was measured by Western blot. (H) The picture of xenograft tumors treated with osimertinib and proguanil were taken after mice were treated for 14 days. (I) Weights of tumors treated with osimertinib and proguanil were measured after 14 days of administration. (J) Western blot analysis of the expression of P62, Beclin‐1, and LC3 in tumor tissues treated with osimertinib and proguanil. (K) T24 cells were treated with osimertinib (1 μM) and rapamycin (0.1 μM) alone or in combination, and the cell viability was measured by colony formation assay. Above: The full view of wells was taken through stereomicroscope. Below: A representative image of the well was taken through an inverted microscope. (L) T24 was treated with osimertinib (2 μM) and rapamycin (0.2 μM) for 24 h, and the expression of p62, Beclin‐1, and LC3 was measured by Western blot. (M) The picture of xenograft tumors treated with osimertinib and rapamycin was taken after mice were treated for 14 days. (N) Weights of tumors treated with osimertinib and rapamycin were measured after 14 days of administration. (O) Western blot analysis of the expression of p62, Beclin‐1, and LC3 in tumor tissues treated with osimertinib and rapamycin. (P) Graphical abstract showed that proguanil or rapamycin enhanced the sensitivity of BC cells to osimertinib. The graphical abstract was designed using the software of Adobe Illustrator CS6. Error bars represent means ± SD from triplicate experiments. Materials and methods are included in Supporting Information (*p < 0.05, **p < 0.01, ***p < 0.001, ^#^ *p* < 0.05, ^##^ *p* < 0.01, ^###^ *p* < 0.001).

Studies have shown that there are four main ways of cell death, which are named apoptosis, ferroptosis, autophagic cell death (ACD), and necrosis.^4^ As EGFR and its downstream signaling pathways are closely related to autophagy regulation in various cancers, and targeting EGFR‐mediated autophagy is a potential strategy for cancer treatment,^2^ we explored whether osimertinib could induce autophagy by inhibiting EGFR and its downstream signaling pathways in BC. Because the conversion of LC3 from LC3‐I to LC3‐II is a specific indicator of the autophagy process, we first detected the changes of LC3‐I/II and found that LC3B‐II/LC3‐I increased. Consistently, osimertinib treatment decreased protein abundance of p62 and upregulated the expression of Beclin‐1, both of which are markers of autophagy (Figure 1C and Figure S2A,B). At the same time, transmission electron microscopy (TEM), monodansylcadaverine (MDC), and immunofluorescence experiments also showed that autophagosomes increased significantly after osimertinib treatment (Figure 1D and Figure S2C,D). These results suggest that autophagy can be induced by osimertinib in BC cells. Interestingly, the inhibition of autophagy by chloroquine (CQ), ammonium chloride (NH_4_Cl), or 3‐methyladenine (3MA) significantly weakened the sensitivity of cells to osimertinib (Figure 1E and Figure S2E), indicating the critical role of autophagy in osimertinib‐inhibited BC cell proliferation.

In recent years, biguanides have attracted much attention because of their superior antitumor activity. Our laboratory explored that the biguanide family drugs metformin, phenformin, and proguanil have excellent bioactivity in BC cells.^2^ Because proguanil has the best antitumor activity among these biguanides, we focused on its antitumor mechanism and observed that proguanil induces autophagic death of BC cells by specific binding to EGFR and inhibiting its expression.^2^ Studies have shown that drug‐induced autophagy can significantly enhance the sensitivity of NSCLC to EGFR‐TKI,^5^ whether the induction of autophagy could enhance the sensitivity of BC cells to osimertinib has not been reported. Therefore, we further investigated whether proguanil could enhance the antitumor activity of osimertinib by inducing autophagy. As expected, the induction of autophagy by proguanil significantly enhanced the sensitivity of cells to osimertinib. Importantly, all of combination index (CI) values were less than 1, indicating a strong synergy of osimertinib and proguanil in BC cells (Figure S3A–D). Consistently, we also observed that the combination of osimertinib and proguanil had better inhibitory effect on cell proliferation than single‐drug treatment (Figure 1F and Figure S3E). To further explore the importance of autophagy in enhancement of proguanil to the inhibitory effect of osimertinib, we examined the changes of autophagy‐related markers in various treatments. The results showed that osimertinib combined with proguanil could significantly downregulate the expression of p62, upregulate the expression of LC3‐II/LC3‐I, Beclin‐1 and increase autophagosome compared with single‐drug treatment (Figure 1D,G and Figure S4A–D), indicating that autophagy might be a critical event in proguanil‐enhanced antitumor effect of osimertinib in BC cells.

To evaluate the antitumor activity of osimertinib in vivo, the model of xenograft tumors was established as described in Supporting Material. As expected, the size of tumors was decreased after osimertinib treatment (Figure 1H and Figure S5A). Interestingly, the combination treatment by osimertinib and proguanil had a better suppression than each of them alone (Figure 1H,I and Figure S5A). The immunohistochemical results of Ki‐67 showed that osimertinib treatment significantly reduced Ki67‐positive cells relative to the control group (Figure S5B). Furthermore, osimertinib plus proguanil treatment further decreased the percentage of Ki67‐positive cells compared with osimertinib‐treated alone (Figure S5B). Additionally, no significant weight loss and no toxicity in liver and kidney tissues were observed (Figure S5C,D). The excised tumors were analyzed by Western blotting, and had a significant decrease in p62 and increase in LC3‐II/LC3‐I and Beclin‐1 in the combined treatment group (Figure 1J and Figure S5E). Therefore, these in vivo data further confirmed the potentiality of osimertinib combined with proguanil to induce autophagy in the treatment of BC.

To verify that proguanil enhanced the effect of osimertinib by inducing autophagy, we examined the combination of osimertinib and the autophagy inducer rapamycin on BC cell death. The results of MTT and clonogenic assay showed that osimertinib and rapamycin had the strong combined effects at all concentrations measured (Figure 1K and Figure S6A–E). Mechanistically, a lower level of p62 and higher level of LC3‐II/LC3‐I and Beclin‐1was observed when treated with both osimertinib and rapamycin as compared to either alone (Figure 1L and Figure S7A,B). Subsequently, MDC stain and immunofluorescence experiments also found that autophagosomes further enhanced after treatment with osimertinib and rapamycin together compared with osimertinib treatment alone (Figure S7C,D). Together, all these data indicated that both proguanil and rapamycin could enhance osimertinib‐induced autophagy to inhibit the proliferation of BC cells, and proguanil or rapamycin have similar mechanisms in enhancing osimertinib sensitivity.

Next, the effects of the autophagy‐inducer rapamycin combined with osimertinib on antitumor activities were also determined in vivo. Our results indicated that rapamycin treatment increased the sensibility of T24 to osimertinib in tumor‐bearing mice, resulting in a significant reduction in tumor size and weight (Figure 1M,N and Figure S8A). Besides, the treatment of rapamycin significantly enhanced the response of osimertinib, resulting in a lower expression of Ki‐67 (Figure S8B). Western blot data also revealed that rapamycin increased the changes in autophagy‐related proteins p62, LC3‐II/LC3‐I, and Beclin‐1induced by osimertinib (Figure 1O and Figure S8C). Importantly, the weight of mice and HE staining of the liver and kidney showed that there were no significant differences between the treatment groups and the vehicle groups (Figure S8D,E). All these data indicated that either proguanil or rapamycin combined with osimertinib had similar effect on autophagy‐related proteins in vivo, suggesting that proguanil‐enhanced osimertinib sensitivity is associated with induction of autophagy.

In conclusion, we have shown that osimertinib induced autophagic death, and the combination of osimertinib with autophagy enhancer including either rapamycin or proguanil could have a better antitumor effect, which may be a promising therapeutic strategy against BC (Figure 1P).

## AUTHOR CONTRIBUTIONS

Di Xiao interpreted results and drafted the manuscript. Simeng Xu conducted the experiments. Xiaochen Zhou and Duo Li prepared and calculated the data. Mei Peng, Xuetong Chu, Zhirong Zhang, and Yan Peng participated in sample and data collection. Alex F. Chen and Xiaoping Yang designed the study, edited the manuscript, and participated in interpretation of the results. All authors read and approved the final version of the manuscript.

## CONFLICT OF INTEREST STATEMENT

The authors declare no conflicts of interest.

## ETHICS STATEMENT

Animal experiment was approved by the Ethics Committee of Hunan Normal University (D2021047).

## Supporting information

Supporting Information

## Data Availability

The data are available from the corresponding author upon reasonable request.
