# Supplementary material for: Synergistic augmentation of osimertinib‐induced autophagic death by proguanil or rapamycin in bladder cancer
Source: MedComm (2020). 2023 Apr 29;4(3):e236. doi: 10.1002/mco2.236 (PMC10148947; doi:10.1002/mco2.236)
Supplement: Supplementary file 1 — Supporting Information [file MCO2-4-e236-s001.docx]

**Supporting information**

**Synergistic Augmentation of Osimertinib-Induced Autophagic Death by Proguanil or Rapamycin in Bladder Cancer**

Di Xiao^1, #^, Simeng Xu ^1, #^, Xiaochen Zhou^1^, Duo Li^1^, Mei Peng^1^, Xuetong Chu^1^, Zhirong Zhang^1^, Yan Peng^1^, Alex F Chen^1, 3*^, Xiaoping Yang^1, 2*^

^1^Key Laboratory of Study and Discovery of Small Targeted Molecules of Hunan Province, Key Laboratory of Chemical Biology & Traditional Chinese Medicine Research of Ministry of Education, Department of Pharmacy, School of Medicine, Hunan Normal University, Changsha, Hunan, China.

^2^Key Laboratory of Protein Chemistry and Developmental Biology of Fish of Ministry of Education, Hunan Normal University, Changsha, Hunan, China.

^3^Institute for Developmental and Regenerative Cardiovascular Medicine, Xinhua Hospital, School of Medicine, Shanghai Jiao Tong University, Shanghai 200092, China. #: These authors contributed equally: Di Xiao, Simeng Xu.

*Corresponding authors

Tel: 86-21-25078910

Fax: 86-21-65076045

E-mail address：chenfengyuan@xinhuamed.com.cn (F. Chen)

Tel/Fax: 86-158-7406-6132

E-mail address：xiaoping.yang@hunnu.edu.cn (X. Yang)

**Contents:** Supplementary Materials and methods (Pages 2-5)

Supplementary Figures (Pages 6-18)

**Materials and methods**

**Cell culture**

Bladder cancer cell lines T24, J82 were obtained from iCell Bioscience Inc (Shanghai, China) and validated for authentication using the short tandem repeat (STR). All cells were performed in 5A (iCell, Shanghai, China) or MEM (Gibco, Grand Island, NY) added in 10% FBS (Hyclone, USA) and 1% penicillin/streptomycin at incubator (37 °C, 5% CO2). Osimertinib (MCE, NJ, USA), Rapamycin (Solarbio, Beijing, China) and proguanil (Selleck, USA) were prepared as a stock solution of 30mM in DMSO.

**MTT assay**

Cells were plated in 96-well plates (6.0 × 10^3^/well). After 12 h, cells were treated or not treated with drugs for 72 h. Subsequently, MTT solution (2 mg/mL, 50 μL) was transferred to wells and incubated for 5 h. Lastly, 150 μL dimethylsulfoxide (DMSO) was added in plates and the absorbance was gauged at 490 nm by using microplate reader (Biotek, SYNERGY HTX, VT, USA).

**Clonogenic assay**

Cells were plated in 24-well plates (2.0 × 10^3^ /well). After 12 h, cells were treated or not treated with drugs and let them to grow for 6–8 days. Subsequently, 10% formaldehyde solution was added to wells to fix the cells. Last, 0.1% crystal violet was transferred to visualize the colonies. Absorbance was gauged at 550 nm with microplate reader (Biotek, USA).

**Western blot**

Protein extracts were resolved through SDS-PAGE which then transferred to PVDF membranes, and probed with primary antibodies (p-EGFR, EGFR, p-AKT, AKT, p-ERK, ERK, Beclin-1, p62 and LC3 were purchased from Cell Signaling Technology (Cell Signaling, Boston, USA).

). Peroxidase conjugated anti-mouse or anti-rabbit antibody was used as secondary antibody and the antigen-antibody reaction was visualized by ChemiDoc system (Bio-Rad, Hercules, CA, USA). The blot band intensities were quantitated by Image J.

**Monodansylcadaverine (MDC) staining**

In order to investigate the autophagy in T24 and J82, MDC kit (Beyotime, China) was utilized according to the manufacturer’s instruction. After cells in each group were treated with drugs for corresponding time, it cleaned with 1× wash buffer. Subsequently, cells were incubated with MDC staining solution in darkness at room temperature for 45 min. After incubation, cells were washed three times with 1× wash buffer. Finally, cells were covered with100 μL of collection buffer and observed under a fluorescence microscope immediately.

**Immunofluorescence**

Cells were seeded on glass coverslips, washed thrice with PBS and fixed in 4% paraformaldehyde solution. After washing with PBS, cells were permeated with 0.2% Triton X-100. After washing again, cells were incubated for 30 min with 4% BSA. The primary antibody was used to incubate cells at 4 °C for overnight. After wash thrice with PBS, DyLight 549 (Proteintech, Chicago, USA) or Alexfluor 488 (Proteintech, USA) labeled secondary antibody was added to glass and incubated for 1 h. Nuclei were stained with DAPI and then fixed with glycerin and photographed under a fluorescence microscope. In Immunofluorescence staining analysis, data were calculated by Image J.

**Subcutaneous xenograft model study**

Four- to six-week-old female BALB/c nude mice (n = 50) were purchased from Hunan SJA Laboratory Animal Co., Ltd (Changsha, China). The study protocol was approved by the Ethics Committee of Hunan Normal University (D2021047). The mice were randomly assigned to groups. Mice were injected subcutaneously with 5x10^6^ T24 suspension to construct xenograft models. Random animals were assigned to each group: (Blank group (mice without tumor), Ctrl group (tumor-bearing mice treated with solvent), Osimertinib (5mg/kg) treatment group, proguanil (10mg/kg) treatment group, Osimertinib (5mg/kg) and proguanil (10mg/kg) combined treatment group, rapamycin (4mg/kg) treatment group, osimertinib (5mg/kg) and rapamycin (4mg/kg) combined treatment group). Tumor volumes were calculated according to the formula: 1/2 long diameter × short diameter^2^. When the tumor grew to 70 - 100 cm^3^, the mice are treated with drugs or solvent (5%DMSO+30%PEG300+5%Tween80+60% double distilled water) through intraperitoneal injection. Tumor volume and mouse weight were gauged every two days. After 14 days of treatment, the mice were sacrificed. The liver and kidney were paraffin-embedded and sectioned and analyzed by HE. Finally, all tumors were kept in formalin for Ki-67 and immunohistochemistry. Investigators were blinded to the group allocation when assessing the results.

**Statistical analysis**

The data of three independent experiments were expressed as mean ± SD. Statistical analysis was performed with SPSS 20.0. A statistical analysis was performed by Student’s t-test. We statistically compared the similar variances between the groups as well. All experiments were repeated at least three times. P < 0.05 was thought as statistically significant.

**Supplementary Figures**


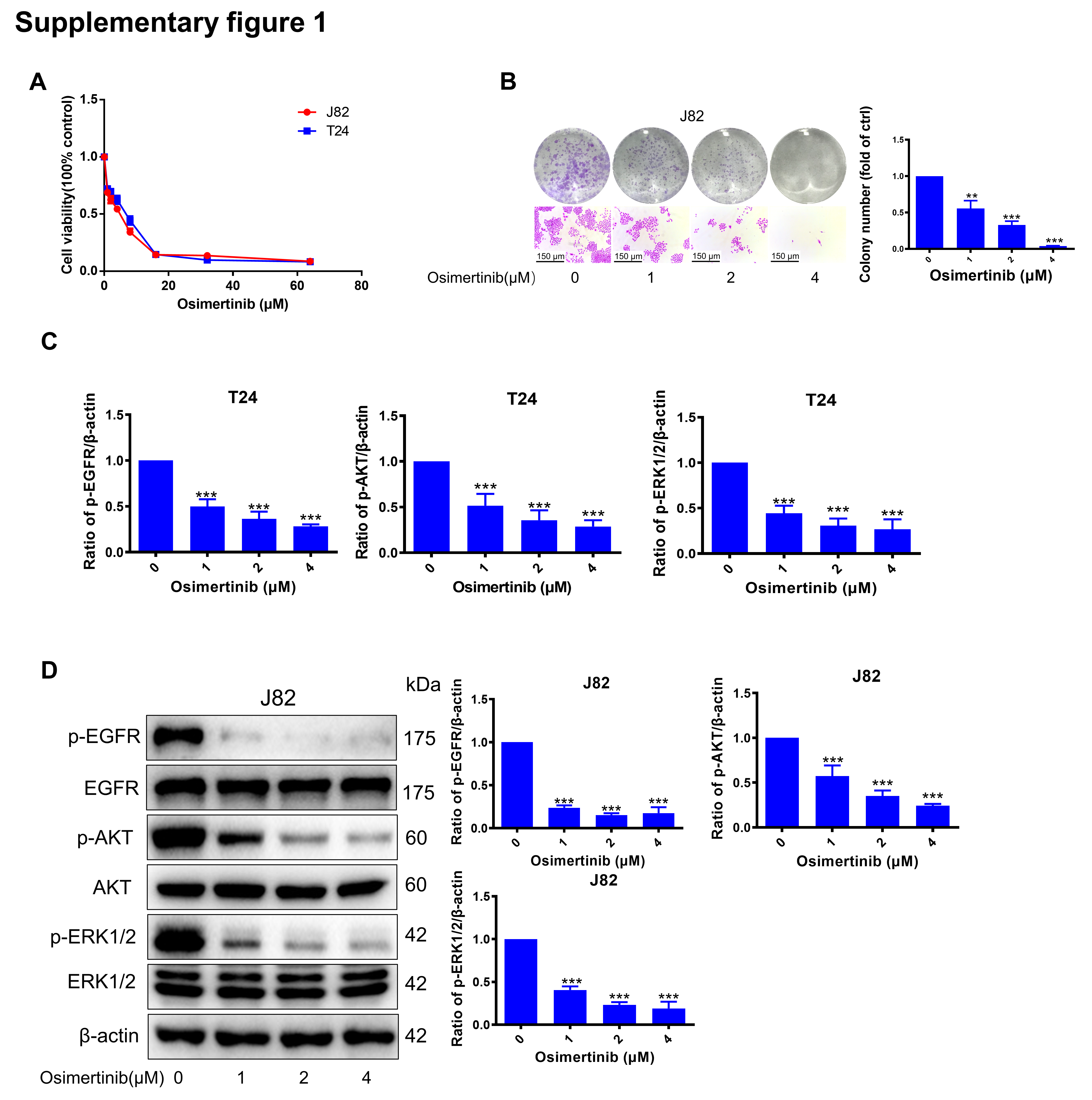


**Supplementary Figure 1. Effect of Osimertinib on the proliferation of bladder cancer cell lines.** (A) J82 and T24 were treated with 0-64μM Osimertinib for 72h, then MTT was added to culture for 4h, and the absorbance at 490 nm was detected using a microplate reader. **(B)** The effect of Osimertinib inhibiting J82 proliferation was evaluated using colony formation assay. Above: The full view of wells was taken through stereomicroscope. Below: A representative image of the well was taken through an inverted microscope. (C**)** Bar graph of p-EGFR, p-AKT and p-ERK in T24 treated with Osimertinib. (D) J82 were treated with Osimertinib for 24h, and the expression of total or phosphorylated EGFR, AKT and ERK were measured by western blot. Data are representative of three independent experiments. Error bars represent means ± SD from triplicate experiments (*P < 0.05, **P < 0.01, ***P < 0.001).


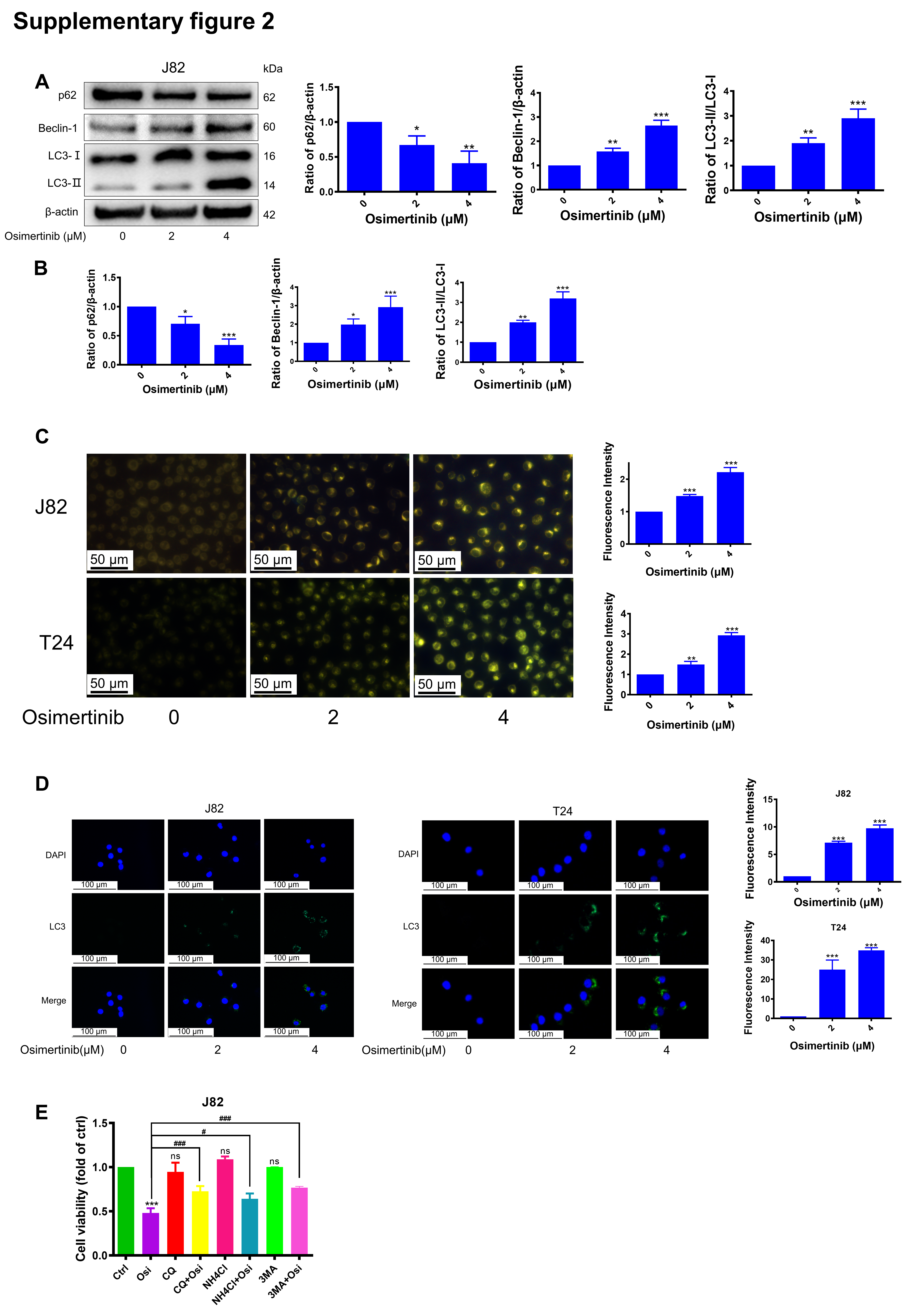


**Supplementary Figure 2. Osimertinib induced autophagy in bladder cancer cell lines.** (A) J82 were treated with Osimertinib for 24h, and the expression of p62, Beclin-1 and LC3 were measured by western blot. (B**)** Bar graph of p62, Beclin-1 and LC3-II/LC3-I in T24 treated with Osimertinib. (C) MDC was used detect autophagosomes in J82 and T24 after treatment with 0-4 μM Osimertinib for 24 h. (D) The expression of LC3 in J82 and T24 treated with 0-4 μM Osimertinib for 24 h was detected by immunofluorescence. (E) J82 were co-treated with 5 μM CQ, 2mM NH4Cl or 5 mM 3MA and 4 μM Osimertinib (Osi) for 72 h, and the cell viability was measured by MTT. Data are representative of three independent experiments. Error bars represent means ± SD from triplicate experiments (*P < 0.05, **P < 0.01, ***P < 0.001, ^#^P < 0.05, ^##^P < 0.01, ^###^P < 0.001).


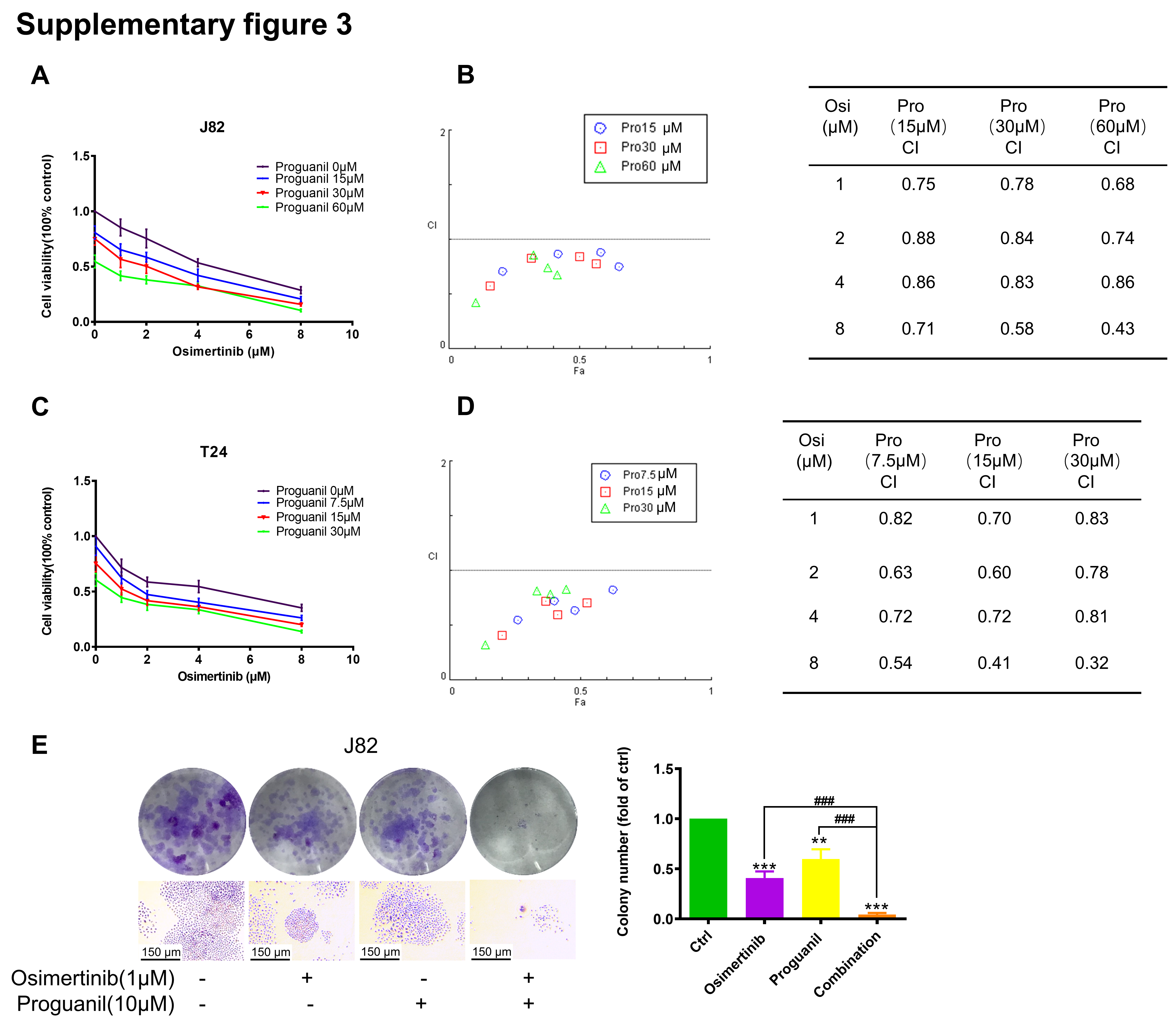


**Supplementary Figure 3. Proguanil enhanced the inhibitory effect of Osimertinib on bladder cancer cell lines.** (A) J82 were treated with Osimertinib and proguanil (Pro) for 72 h, the combined effect of Osimertinib and proguanil was detected by MTT. (B) Combination index (CI) among the combinations of two drugs was calculated using CompuSyn software. if CI > 1, it denotes antagonism; if CI < 1, it denotes synergism. CI values in all of combinations were less than 1, indicating synergism. (C) T24 were treated with Osimertinib and proguanil for 72 h, the combined effect of Osimertinib and proguanil was detected by MTT. (D) Combination index (CI) among the combinations of two drugs was calculated using CompuSyn software. if CI > 1, it denotes antagonism; if CI < 1, it denotes synergism. CI values in all of combinations were less than 1, indicating synergism. (E**)** J82 were treated with Osimertinib (1μM) and proguanil (10μM) alone or in combination, and the cell viability was measured by colony formation assay. Above: The full view of wells was taken through stereomicroscope. Below: A representative image of the well was taken through an inverted microscope. Data are representative of three independent experiments. Error bars represent means ± SD from triplicate experiments (*P < 0.05, **P < 0.01, ***P < 0.001, ^#^P < 0.05, ^##^P < 0.01, ^###^P < 0.001).


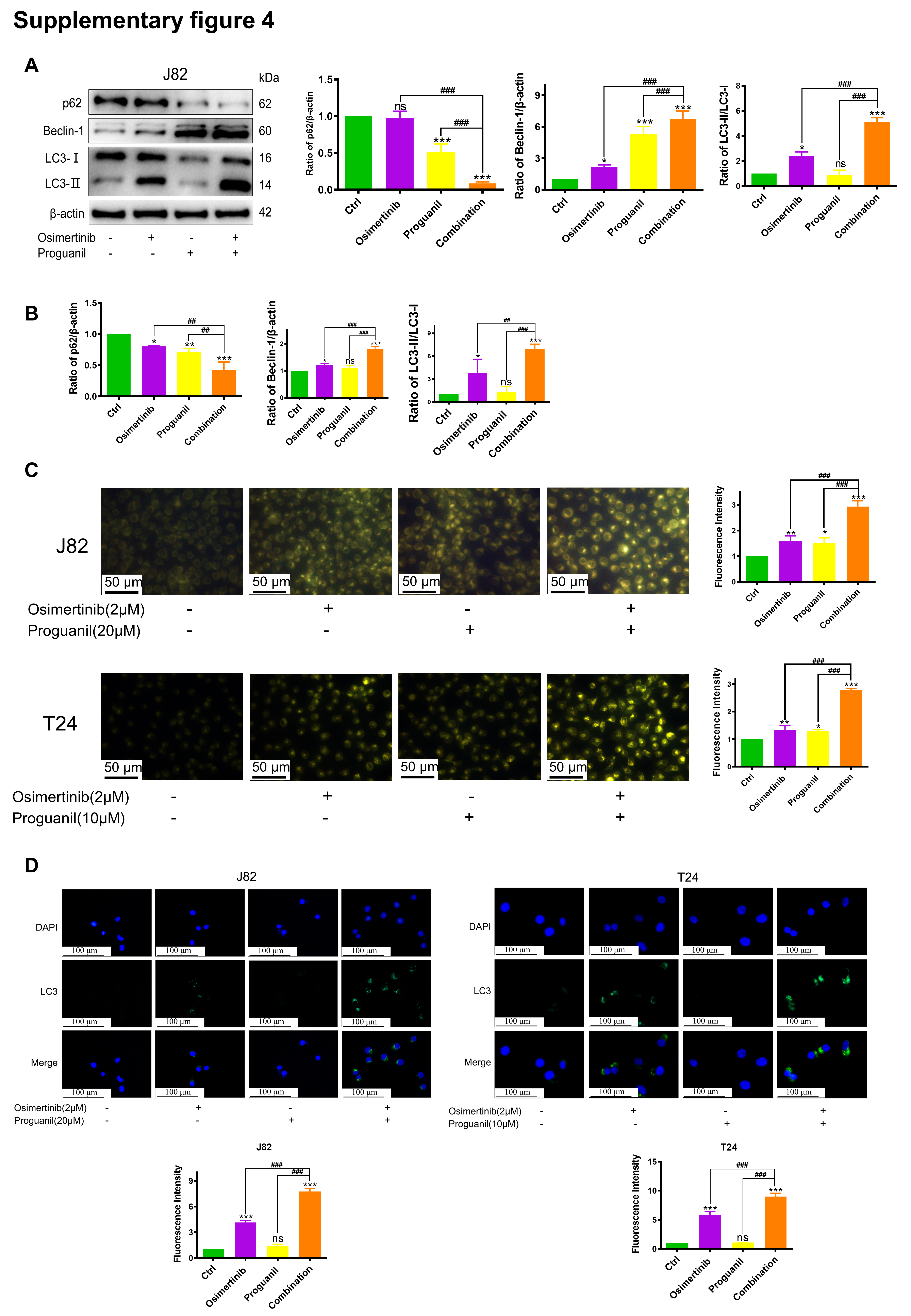


**Supplementary Figure 4. Osimertinib and proguanil synergistically induced autophagy in bladder cancer cell lines.** (A) J82 were treated with Osimertinib (2μM) and proguanil(20μM) for 24h, and the expression of p62, Beclin-1 and LC3 were measured by western blot. (B**)** Bar graph of p62, Beclin-1 and LC3-II/LC3-I in T24 treated with Osimertinib and proguanil. (C) MDC was used detect autophagosomes in J82 and T24 after treatment with Osimertinib and proguanil for 24 h. (D) The expression of LC3 in J82 and T24 treated with Osimertinib and proguanil for 24 h was detected by immunofluorescence. Data are representative of three independent experiments. Error bars represent means ± SD from triplicate experiments (*P < 0.05, **P < 0.01, ***P < 0.001, ^#^P < 0.05, ^##^P < 0.01, ^###^P < 0.001).


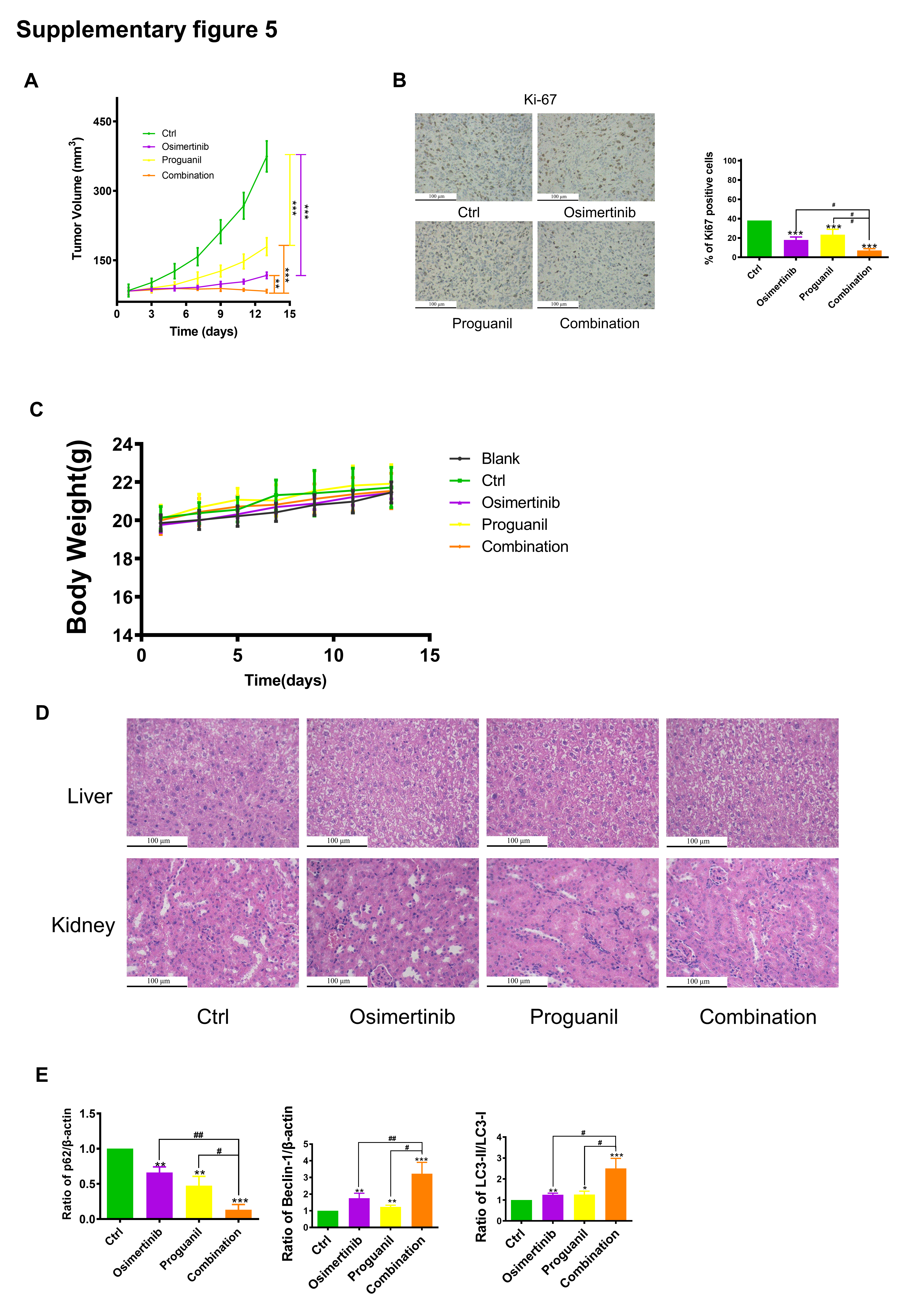


**Supplementary Figure 5. Proguanil enhanced the inhibition of Osimertinib in nude mice xenografts.** (A) The tumor volume of each mouse was measured every two days, and the mean volume of each group tumor was calculated to create the figure. (B) Ki-67 was used to analyze the proliferation of xenograft tumor. (C) Changes of each group mice weight. (D) HE staining of liver and kidney organs of each group mice. (E**)** Bar graph of p62, Beclin-1 and LC3-II/LC3-I in tumor tissues treated with Osimertinib and proguanil. Data are representative of three independent experiments. Error bars represent means ± SD from triplicate experiments (*P < 0.05, **P < 0.01, ***P < 0.001, ^#^P < 0.05, ^##^P < 0.01, ^###^P < 0.001).


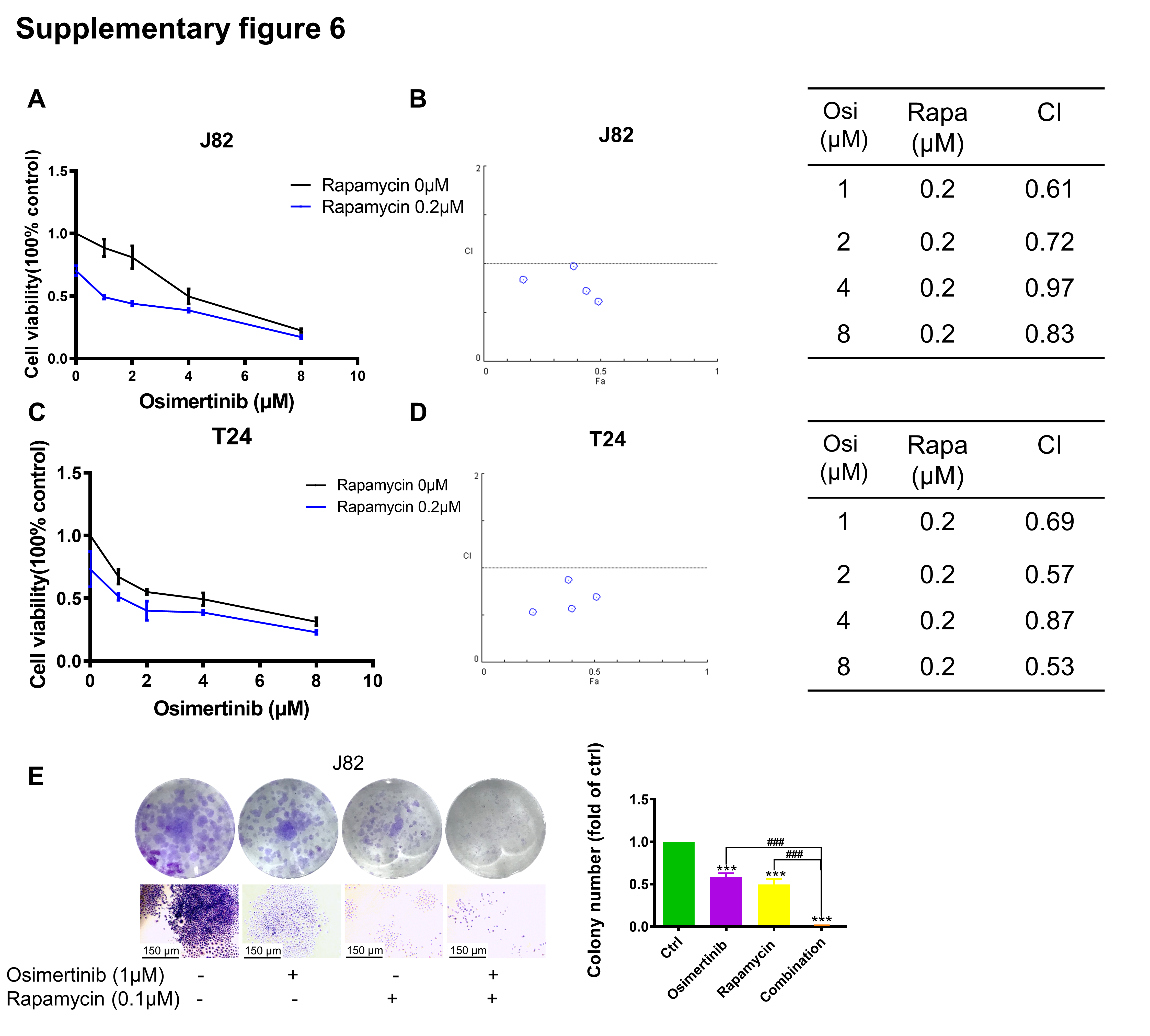


**Supplementary Figure 6. Rapamycin enhanced the inhibitory effect of Osimertinib on bladder cancer cell lines.** (A) J82 were treated with Osimertinib and rapamycin (Rapi) for 72 h, the combined effect of Osimertinib and rapamycin was detected by MTT. (B) Combination index (CI) among the combinations of two drugs was calculated using CompuSyn software. if CI > 1, it denotes antagonism; if CI < 1, it denotes synergism. CI values in all of combinations were less than 1, indicating synergism. (C) T24 were treated with Osimertinib and rapamycin for 72 h, the combined effect of Osimertinib and rapamycin was detected by MTT. (D) Combination index (CI) among the combinations of two drugs was calculated using CompuSyn software. if CI > 1, it denotes antagonism; if CI < 1, it denotes synergism. CI values in all of combinations were less than 1, indicating synergism. (E**)** J82 were treated with Osimertinib (1μM) and rapamycin (0.1μM) alone or in combination, and the cell viability was measured by colony formation assay. Above: The full view of wells was taken through stereomicroscope. Below: A representative image of the well was taken through an inverted microscope. Data are representative of three independent experiments. Error bars represent means ± SD from triplicate experiments (*P < 0.05, **P < 0.01, ***P < 0.001, ^#^P < 0.05, ^##^P < 0.01, ^###^P < 0.001).


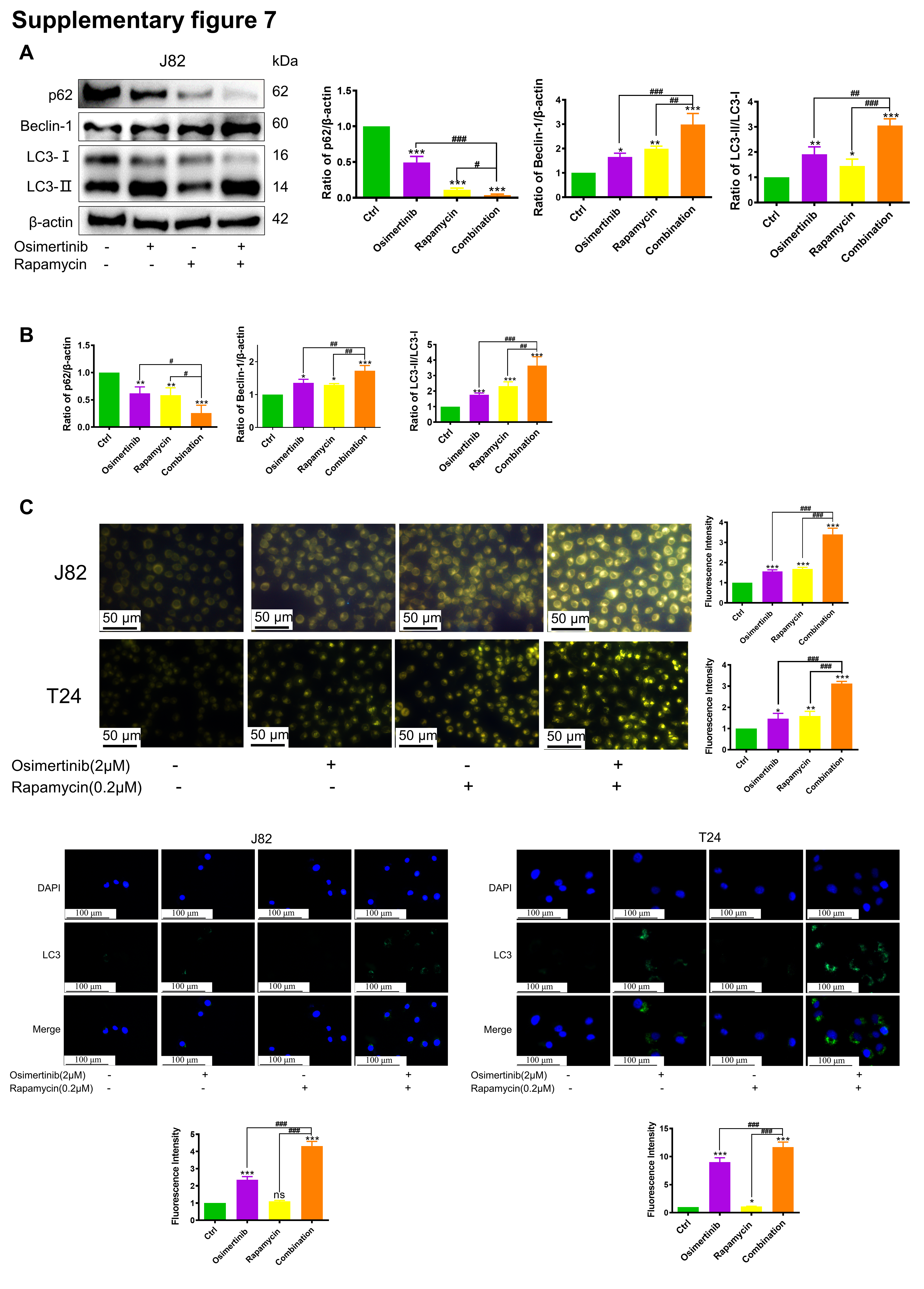


**Supplementary Figure 7. Osimertinib and Rapamycin synergistically induced autophagy in bladder cancer cell lines.** (A) J82 were treated with Osimertinib (2μM) and rapamycin (0.2μM) for 24h, and the expression of p62, Beclin-1 and LC3 were measured by western blot. (B**)** Bar graph of p62, Beclin-1 and LC3-II/LC3-I in T24 treated with Osimertinib (2μM) and rapamycin (0.2μM). (C) MDC was used detect autophagosomes in J82 and T24 after treatment with Osimertinib (2μM) and rapamycin (0.2μM) for 24 h. (D) The expression of LC3 in J82 and T24 treated with Osimertinib (2μM) and rapamycin (0.2μM) for 24 h was detected by immunofluorescence. Data are representative of three independent experiments. Error bars represent means ± SD from triplicate experiments (*P < 0.05, **P < 0.01, ***P < 0.001, ^#^P < 0.05, ^##^P < 0.01, ^###^P < 0.001).


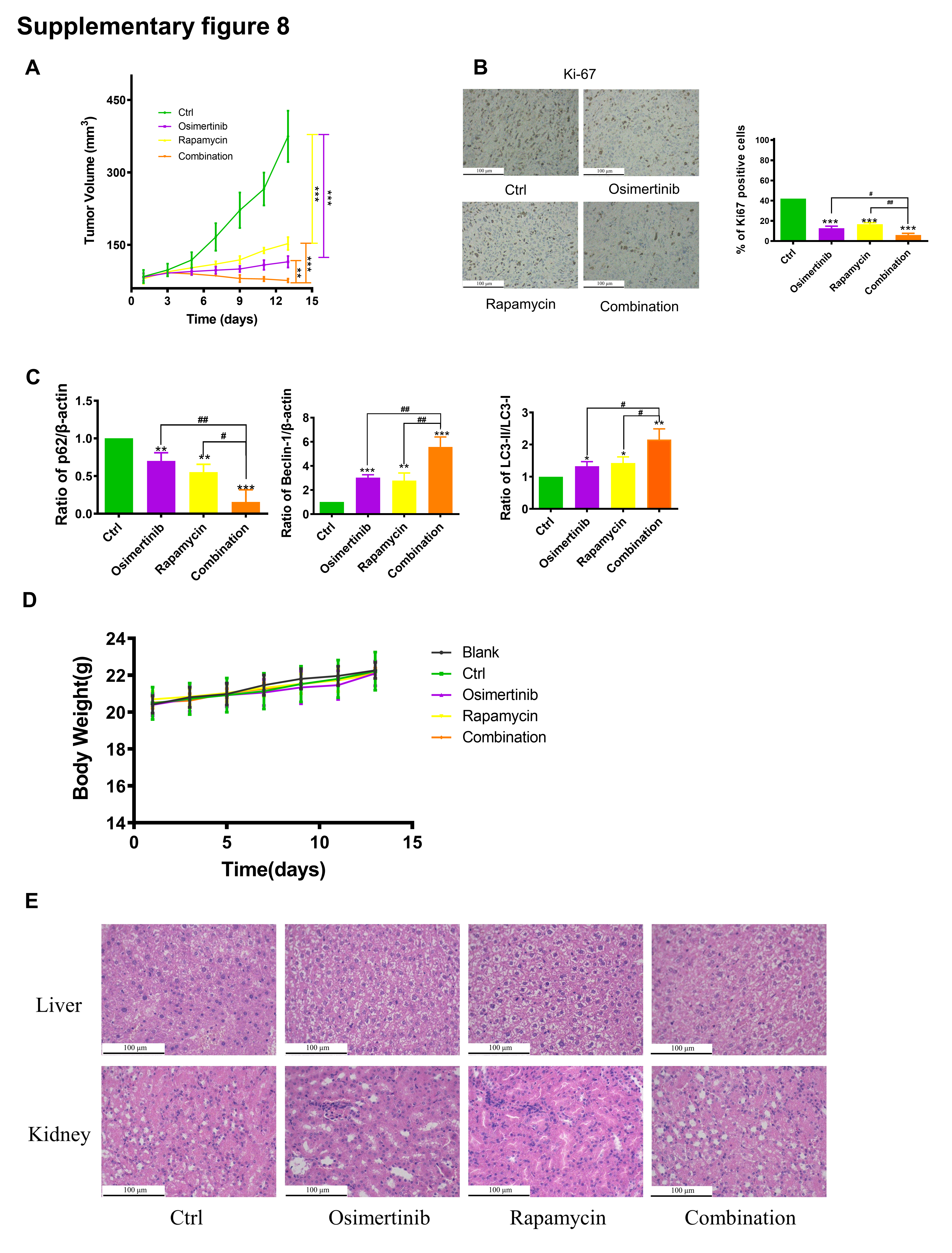


**Supplementary Figure 8.** Rapamycin enhanced the inhibition of Osimertinib in nude mice xenografts. (A) The tumor volume of each mouse was measured every two days, and the mean volume of each group tumor was calculated to create the figure. (B) Ki-67 was used to analyze the proliferation of xenograft tumor. (C**)** Bar graph of p62, Beclin-1 and LC3-II/LC3-I in tumor tissues treated with Osimertinib and Rapamycin. (D) Changes of each group mice weight. E) HE staining of liver and kidney organs of each group mice. Data are representative of three independent experiments. Error bars represent means ± SD from triplicate experiments (*P < 0.05, **P < 0.01, ***P < 0.001, ^#^P < 0.05, ^##^P < 0.01, ^###^P < 0.001).
